# Genome-wide association analysis to delineate high-quality SNPs for seed micronutrient density in chickpea (*Cicer arietinum* L.)

**DOI:** 10.1038/s41598-022-14487-1

**Published:** 2022-09-05

**Authors:** Humara Fayaz, Sandhya Tyagi, Aijaz A. Wani, Renu Pandey, Sabina Akhtar, Mohd Ashraf Bhat, Annapurna Chitikineni, Rajeev Kumar Varshney, Mahendar Thudi, Upendra Kumar, Reyazul Rouf Mir

**Affiliations:** 1 Division of Genetics and Plant Breeding, Faculty of Agriculture (FoA), Sher-e-Kashmir University of Agricultural Sciences & Technology (SKUAST)-Kashmir, Wadura Campus, Sopore, India; 2 Cytogenetics and Reproductive Biology Laboratory, Department of Botany, University of Kashmir, Srinagar, India; 3 Division of Plant Physiology, Indian Agricultural Research Institute (IARI), New Delhi, India; 4 College of Education, American University in the Emirates, Dubai, UAE; 5 Center of Excellence in Genomics & Systems Biology (CEGSB), Iinternational Crops Research Institute for the Semi-Arid Tropics (ICRISAT), Patancheru, Hyderabad, Telangana India; 6 State Agricultural Biotechnology Centre, Crop & Food Innovation Centre, Food Futures Institute, Murdoch University, Murdoch, WA Australia; 7 Department of Agricultural Biotechnology and Biotechnology, Rajendra Prasad Central Agricultural University, Pusa, Samasthipur India; 8 University of Southern Queensland (USQ), Toowoomba, Australia; 9 Department of Molecular Biology, Biotechnology and Bioinformatics, College of Biotechnology, CCS Haryana Agricultural University, Hisar, 125004 India

**Keywords:** Genetics, Plant sciences

## Abstract

Chickpea is the most important nutrient-rich grain legume crop in the world. A diverse core set of 147 chickpea genotypes was genotyped with a Axiom(®)50K CicerSNP array and trait phenotyped in two different environments for four seed micronutrients (Zn, Cu, Fe and Mn). The trait data and high-throughput 50K SNP genotypic data were used for the genome-wide association study (GWAS). The study led to the discovery of genes/QTLs for seed Zn, Cu, Fe and Mn, concentrations in chickpea. The analysis of seed micronutrient data revealed significant differences for all four micronutrient concentrations (P ≤ 0.05). The mean concentrations of seed Zn, Cu, Fe and Mn pooled over the 2 years were 45.9 ppm, 63.8 ppm 146.1 ppm, and 27.0 ppm, respectively. The analysis of results led to the identification of 35 SNPs significantly associated with seed Zn, Cu, Fe and Mn concentrations. Among these 35 marker-trait associations (MTAs), 5 were stable (consistently identified in different environments), 6 were major (explaining more than 15% of the phenotypic variation for an individual trait) and 3 were both major and stable MTAs. A set of 6 MTAs, MTAs (3 for Mn, 2 for Fe, and 1 for Cu) reported by us during the present study have been also reported in the same/almost same genomic regions in earlier studies and therefore declared as validated MTAs. The stable, major and validated MTAs identified during the present study will prove useful in future chickpea molecular breeding programs aimed at enhancing the seed nutrient density of chickpea.

## Introduction

Chickpea (*Cicer arietinum* L.; 2*n* = 2*x* = 16) is one of the most important grain legume crops in the world and is well known for its health and nutritional benefits. As far as importance is concerned, the crop holds second place with respect to its importance and third place in production among the pulses grown worldwide^1,2^. The world population is growing at a very fast rate and is believed to escalate the mark of 9 billion by 2050^3,4^. To meet the dietary demands of this expanding population, it is important to develop nutrient-dense crops for the poorer sections of society. Micronutrient elements such as zinc (Zn), iron (Fe), copper (Cu), magnesium (Mg), phosphorous (P), potassium (K) and manganese (Mn) play vital roles in the metabolism and physiological processes of both plants and humans^5–7^. The deficiency of these nutrient elements is caused mainly by non-availability or their low concentrations in the staple diets, resulting in serious health issues for billions of people world-wide^8,9^. The dietary importance of chickpea is well known across the global human population^10^. As far as bio-fortification through marker-assisted breeding (MAB) is concerned, the complex genetic inheritance pattern and the gene regulatory functions of micronutrients such as Zn and Fe concentrations are of paramount importance. These micronutrients in chickpea are complex quantitative in nature, showing minor genotypic effects but a higher magnitude of environmental effects^11^. The same was also validated in another study where it was demonstrated that genotypic contribution to total variability is limited compared to year and location effects^12^. The concentration of these minerals is also influenced by the seed size, seed type (Desi vs Kabuli) and some time by seed coat color^10,13,14^.

Quantitative trait loci (QTLs) for Fe and Zn were reported using a genotyping by sequencing (GBS) approach^15^. Biofortification has an outstanding role in increasing the concentration of micronutrients and their bioavailability in crop plants, especially in grain legumes, which have considerable importance in the human diet^16^. Regardless of the importance of the process of biofortification, there has been limited research on the development of micronutrient-rich crops over the last half century^17^. As far as research on biofortification in legumes is concerned, its focus during the last few decades has been limited to micronutrients such as seed Zn and seed Fe concentrations only and more recently, the focus has been shifted to nutrients such as Cu, P and K^5–7^.

Genome-wide association studies (GWASs) are considered one of the paramount and emerging approaches of gene/QTL discovery for the genetic dissection of quantitative traits, including seed micronutrients, in crop plants^18,19^. GWAS serves as an alternative method of gene discovery to QTL mapping that involves the use of biparental mapping and eradicates several drawbacks of the latter in the identification of QTLs/genes^20–23^. GWASs have been successfully used in the discovery of genes/QTLs for a variety of traits, including seed micronutrients, in cereals^24^ as well as legumes^25,26^. Association-mapping studies have brought insights into how to improve the concentration of mineral nutrients in crops such as rice^27^, barley^18^, field pea^28^, common bean^29^ and chickpea^5–7,30^. Recently, in the context of climate change, efforts were also made to identify the effect of drought and heat on the accumulation of micronutrients, and MTAs were also reported^31^. For GWAS, several genotyping platforms based on SNPs have been developed over the years and have been used successfully for the identification of genes for different traits in crop plants^32^. The availability of the draft genome sequence of chickpea^33^ and large-scale resequencing efforts^34–36^ have led to the discovery of large-scale SNP markers for the development of SNP genotyping platforms such asAxiom^®^CicerSNP Array^37^^.^

In the present study, we report the marker trait associations (MTAs) for micronutrients using the core set of germplasm and the Axiom^®^CicerSNP Array. The results of the present study will encourage the effective utilization of chickpea genetic resources, opening possibilities for biofortification programs in chickpea in the near future.

## Results

### Variability for seed Zn, Cu, Fe and Mn concentrations

**Figure 1:**
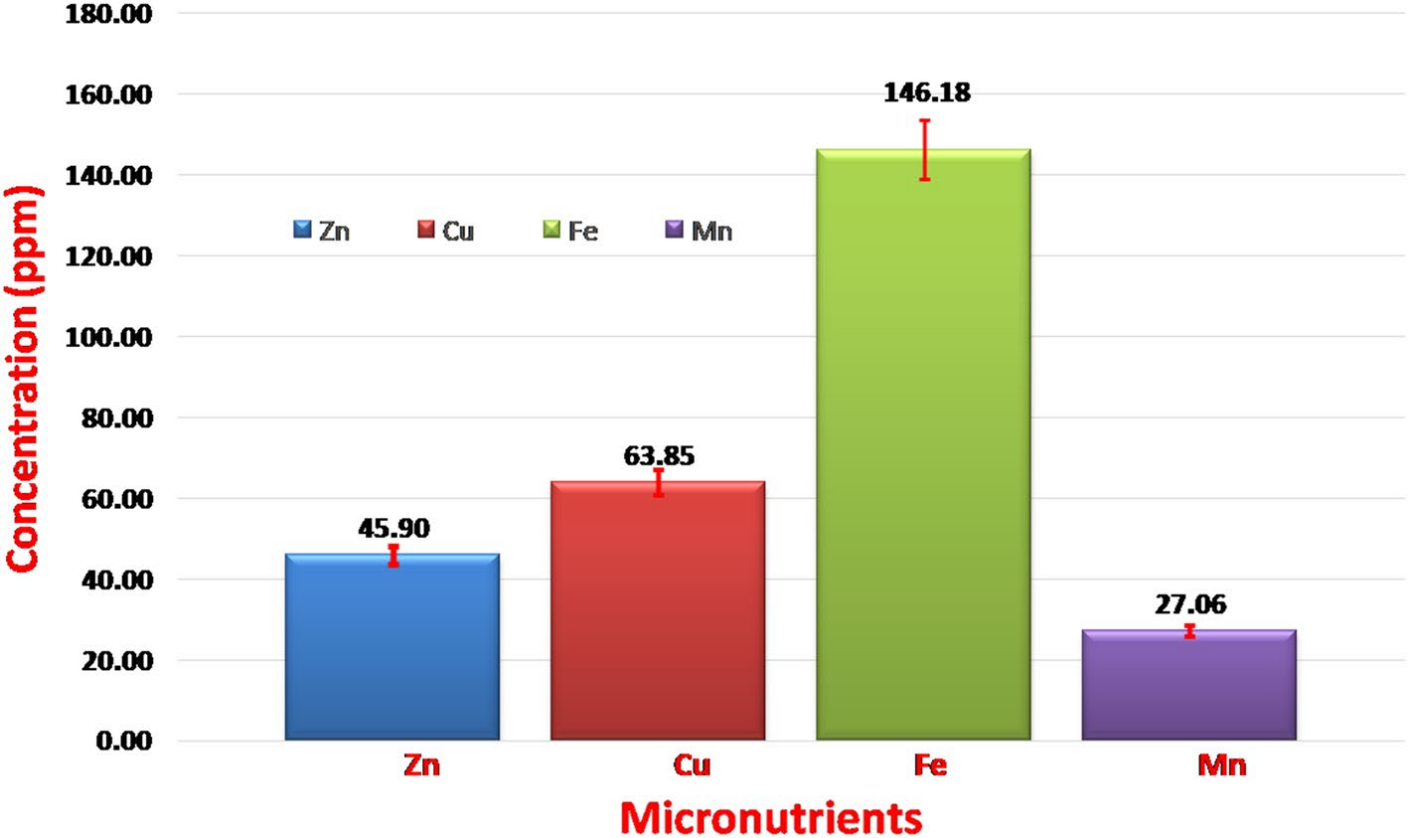
The frequency distribution of trait data of four micronutrients (Zn, Cu, Fe and Mn) pooled over two environments. The lines on each plot shows the error bars. The figure shows wide variation shown by seed Fe concentrations, followed by seed Cu, Zn and Mn concentrations.

We estimated the concentrations of micronutrients such as Zn, Cu, Fe and Mn in all 147 chickpea seed samples based on 2017–2018 and 2018–2019 as well as pooled samples from both years. We observed a large variation in the concentration of the estimated micronutrients between the years as well as the pooled samples. Based on the samples obtained during 2017–2018, the Zn concentration ranged from 14.56 to 204.39 ppm with a mean of 45.31 ppm, the Cu concentration ranged from 4.23 to 185.47 ppm (mean 60.99 ppm), the Fe concentration ranged from 79.36 to 198.26 ppm (mean 143.24 ppm) and the Mn concentration ranged from 9.07 to 54.29 ppm (mean 25.62 ppm) (Table 1). Similarly, analysis of seed micronutrient concentrations in Year II revealed that the Zn concentration ranged from 9.36 to 205.86 ppm (mean 46.58 ppm), Cu concentration ranged from 3.66 to 190.36 ppm (mean 66.81 ppm), Fe concentration ranged from 100.36 to 127.2 ppm (mean 132.29 ppm) and Mn concentration ranged from 4.86 to 122.86 ppm (mean 28.68 ppm) (Table 1). The mean values of Zn, Cu, Fe and Mn concentrations in Environment III (pooled data) are presented in Fig. 1. Levene’s test conducted for the trait data of two different environments of seed Zn, Cu, Fe and Mn concentrations revealed non-significant differences in variances (P for Zn = 0.48; P for Cu = 0.99; P for Fe = 0.99 and P value for Mn = 0.98) of the trait data of two environments/experiments. Thus, the results indicated that the variances are homogeneous between the two experiments. Combined ANOVA indicated significant interactions (P ≤ 0.5%) between genotype and environment (year) for all four micronutrients (Zn, Cu, Fe and Mn). The mean micronutrient concentrations of the genotypes varied significantly. The maximum coefficient of variation was observed for seed Mn (2.68%), followed by Zn (1.58%), Cu (1.40%) and Fe (0.52%) (Table 2).

**Table 1:** Trait variation in the concentrations of seed Zn, Cu, Fe and Mn concentration in the chickpea core set evaluated during the year 2017–2018, year 2018–2019 and data pooled over environments.

Value	Zn (ppm)			Cu (ppm)			Fe (ppm)			Mn (ppm)		
	Year 2017	Year 2018	Pooled data	Year 2017	Year 2018	Pooled data	Year 2017	Year 2018	Pooled data	Year 2017	Year 2018	Pooled data
Min.	14.5	9.3	11.9	4.2	3.6	3.9	79.3	100.3	97.1	9.0	4.8	10.1
Max.	204.3	205.8	205.1	185.4	190.3	183.9	198.2	127.2	194.5	54.2	122.8	79.0
Avg ± SD	45.3 ± 26.0	46.5 ± 27.4	45.9 ± 26.5	60.9 ± 40.3	66.8 ± 41.8	63.8 ± 40.2	143.2 ± 29.4	132.2 ± 29.0	146.1 ± 22.4	25.6 ± 8.9	28.6 ± 17.7	27.0 ± 10.3

**Table 2:** Combined Analysis of Variance (ANOVA) conducted for chickpea seed micronutrient (Zn, Cu, Fe and Mn) concentrations. The table shows the variation contributed by different sources/factors for seed micronutrient data collected over different years.

Source of variation	Degrees of freedom	Zn		Cu		Fe		Mn	
		Mean sum of squares	F-stat	Mean sum of squares	F-stat	Mean sum of squares	F-stat	Mean sum of squares	F-stat
Environment (E)	1	366.6	296.6**	8116.6	5212.9**	25,143.0	1654.0**	887.0	710.6**
Genotype (G)	146	4215.0	3410.2**	9733.7	6251.4**	12,129.1	8100.9**	642.7	514.9**
G × E	146	81.9	66.3**	417.0	267.8**	52,555	3369.0**	547.3	438.5**
Error	588	1.2	1	1.5	1	1.5	1	1.2	1
Coefficient of variation		1.58%		1.40%		0.52%		2.68%	

**Denotes that values are statistically significant at p-value of less than 0.01.

### Single nucleotide polymorphism (SNP) distribution on the chickpea genome

**Figure 2:**
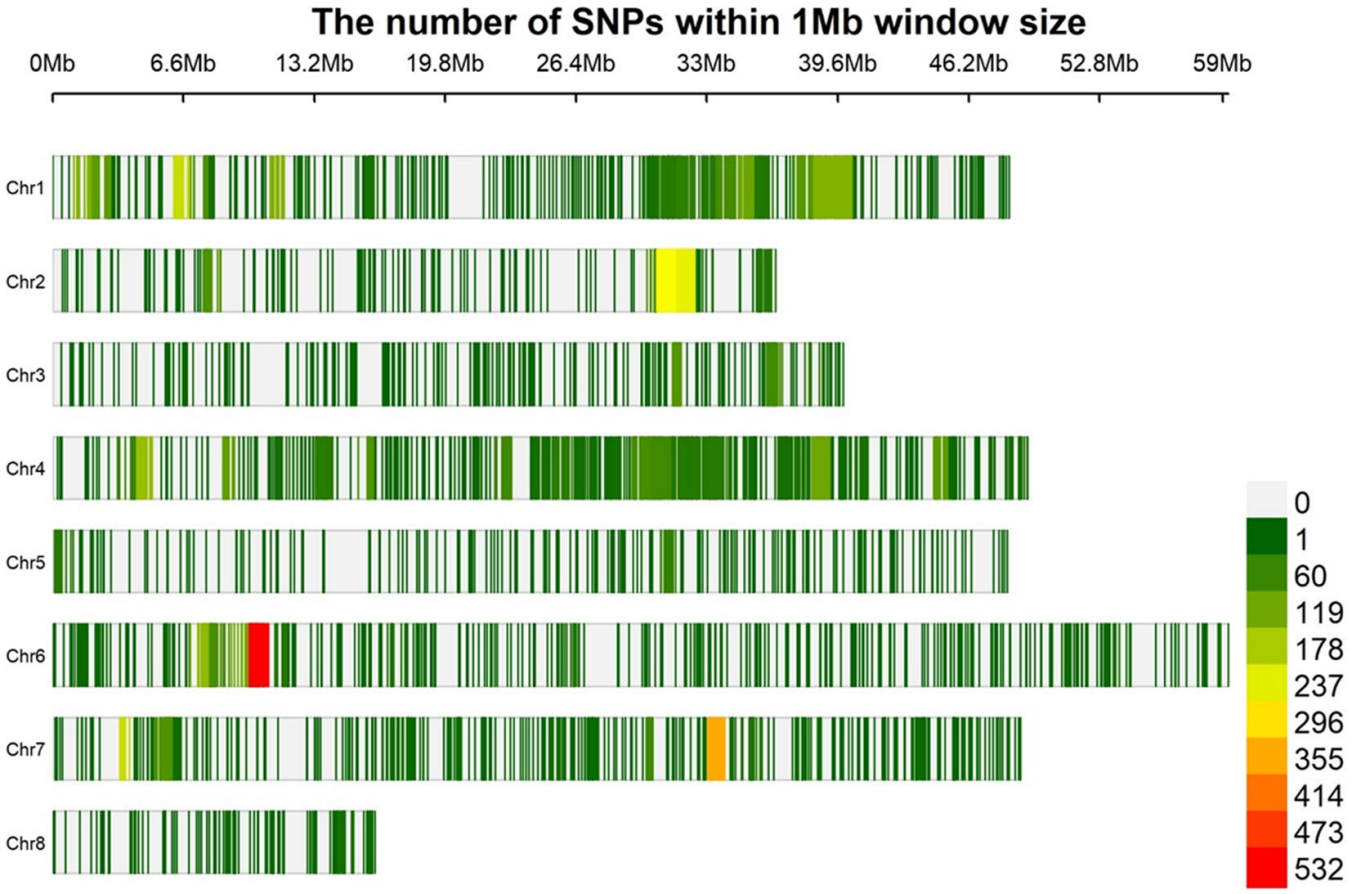
Distribution of 7277 high-quality SNPs over 8 different chickpea chromosomes. The figure shows the number of SNPs within 1 Mb window and reflects the SNP density on each chromosomes used during the present study for GWAS for micronutrients.

We genotyped the 147 chickpea genotypes with an Axiom^®^*CicerSNP* Array comprising 50,590 SNPs, and SNPs were called following the Axiom best practice workflow^29^. We excluded SNPs with ambiguous bases as well as all loci with more than 10% missing data. Furthermore, we also excluded monomorphic SNPs and SNPs with a PIC value < 0.349%. As a result, we obtained a total of 7277 high-quality SNPs that were used for further analysis (Table 3, Fig. 2). On average, we identified 5186 SNPs per pseudomolecule, while the number of SNPs on each pseudomolecule ranged from 119 (Ca8) to 1686 (Ca1) (Table 3). The PIC values of SNPs varied from 0.364 to 0.368. Among the 7277 SNP markers, 920 (12.64%) had a maximum PIC value of 0.375, whereas 200 (2.74%) had a minimum PIC value of 0.350, while the majority of the SNPs 6,157 (84.60%) had a PIC value between 0.350 and 0.375.

**Table 3:** Distribution of SNPs on eight different pseudomolecules/chromosomes in chickpea and their polymorphism information content (PIC). The table also shows the number of SNPs available on each chromosomes/linkage group and the number of SNPs used for conducting marker-trait associations during the present study.

Pseudomolecule (linkage group)	Number of SNPs (used for analysis)	Pseudomolecule (linkage group) (bp)	Mean of SNPs/Mbp	PIC value range (Mean)
Ca1	7313 (1686)	4,82,94,326.00	1517.219917	0.349–0.375 (0.364)
Ca2	3569 (843)	3,66,27,078.00	975.136612	0.349–0.375 (0.365)
Ca3	2724 (413)	3,99,87,104.00	682.706766	0.349–0.375 (0.363)
Ca4	14,180 (1455)	4,91,79,665.00	2887.983707	0.349–0.375 (0.365)
Ca5	2627 (313)	4,81,54,507.00	546.153846	0.349–0.375 (0.366)
Ca6	5121 (1331)	5,94,20,319.00	862.121212	0.349–0.375 (0.367)
Ca7	4364 (1117)	4,89,52,158.00	892.433537	0.349–0.375 (0.368)
Ca8	1591 (119)	1,64,71,619.00	970.121951	0.351–0.375 (0.365)
Total	41,489 (7277)

### Diversity analysis

**Figure 3:**
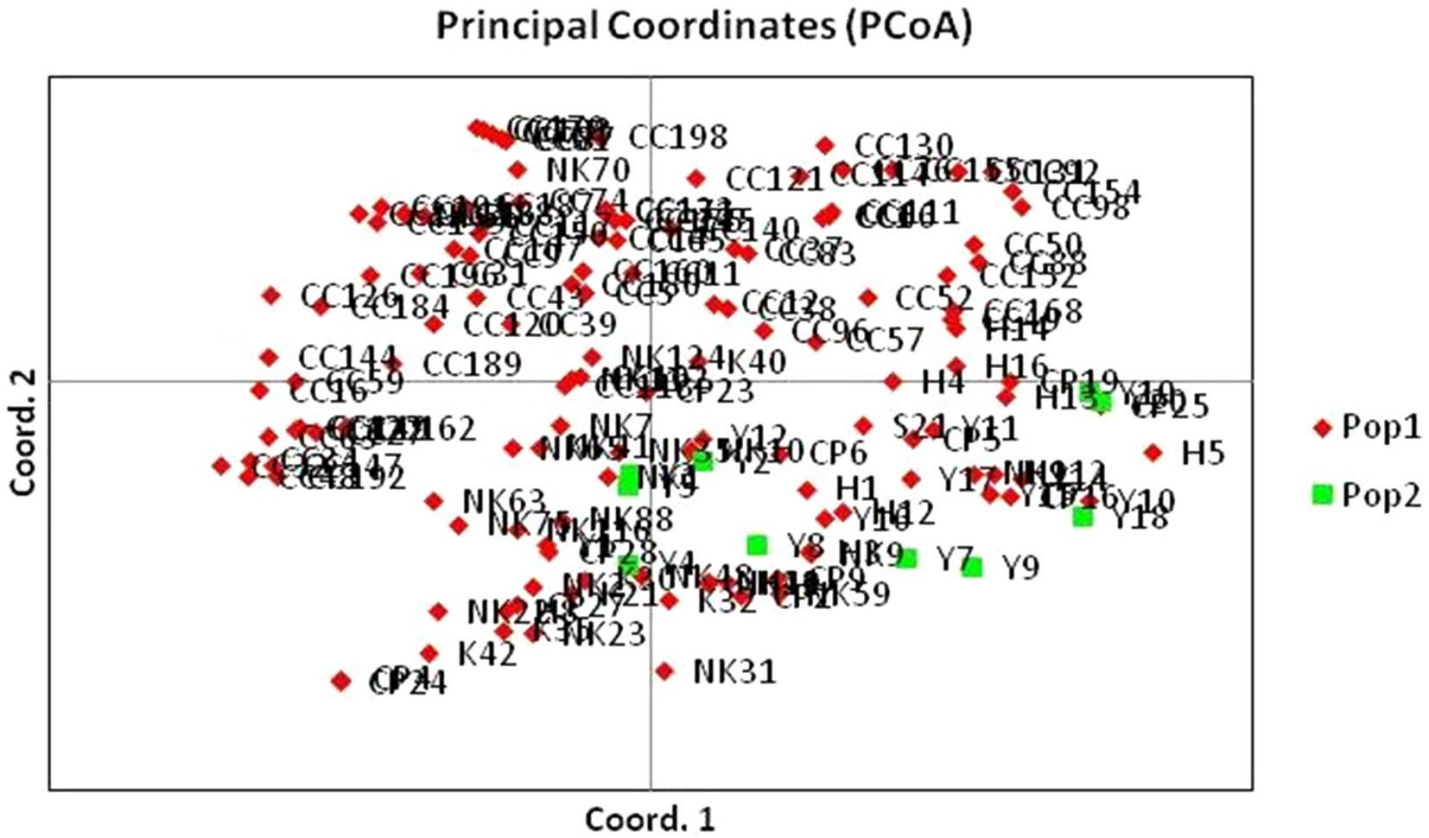
PCoA showing clustering of different chickpea genotypes into different co-ordinates. The genotypes highlighted with different colors (black versus green) shows two different sub-populations.

We identified two subpopulations in the chickpea core set based on principal component analysis (PCA) (Fig. 3). Analysis of molecular variance (AMOVA) among the two subpopulations identified based on PCA indicated that the major source of variance was the genotypes, not the populations. The variation of 7% was due to populations (between populations), and 93% variation was within the population, i.e., due to genotype alone (Supplementary Table S1). The maximum number of private alleles (398) was present in the indigenous population, exhibiting that the indigenous genotypes are diverse in comparison to the exotic ones (Supplementary Table S2). A diversity analysis study was conducted to estimate important diversity parameters of the core set. The summary of the results of diversity statistics presented in Table 4 shows that population-1 is more diverse than population-2, with higher values for diversity parameters such as gene diversity “He” (major index of diversity).

**Table 4:** Summary of the different genetic diversity parameters used to infer the extent of genetic diversity available in different chickpea sub-populations.

Population		N	Na	Ne	I	Ho	He	F	%P
**Mean and SE over loci for each pop**									
Pop1	Mean	122.285	2.000	1.927	0.673	0.199	0.481	0.587	100.00
	SE	0.176	0.000	0.001	0.000	0.002	0.000	0.005	
Pop2	Mean	8.984	1.934	1.670	0.549	0.154	0.377	0.566	93.46
	SE	0.017	0.003	0.004	0.002	0.003	0.002	0.007	
**Grand mean and SE over loci and pops**									
Total	Mean	65.635	1.967	1.799	0.611	0.176	0.429	0.577	96.73
	SE	0.478	0.001	0.002	0.001	0.002	0.001	0.004	3.27

*N* sample size, *Na* number of different alleles, *Ne* number of effective alleles, *I* Shannon’s information index, *Ho* observed heterozygosity, *He* expected heterozygosity, *%P* variation explained, *SE* standard error.

### Significant marker-trait associations for micronutrient concentrations

**Figure 4:**
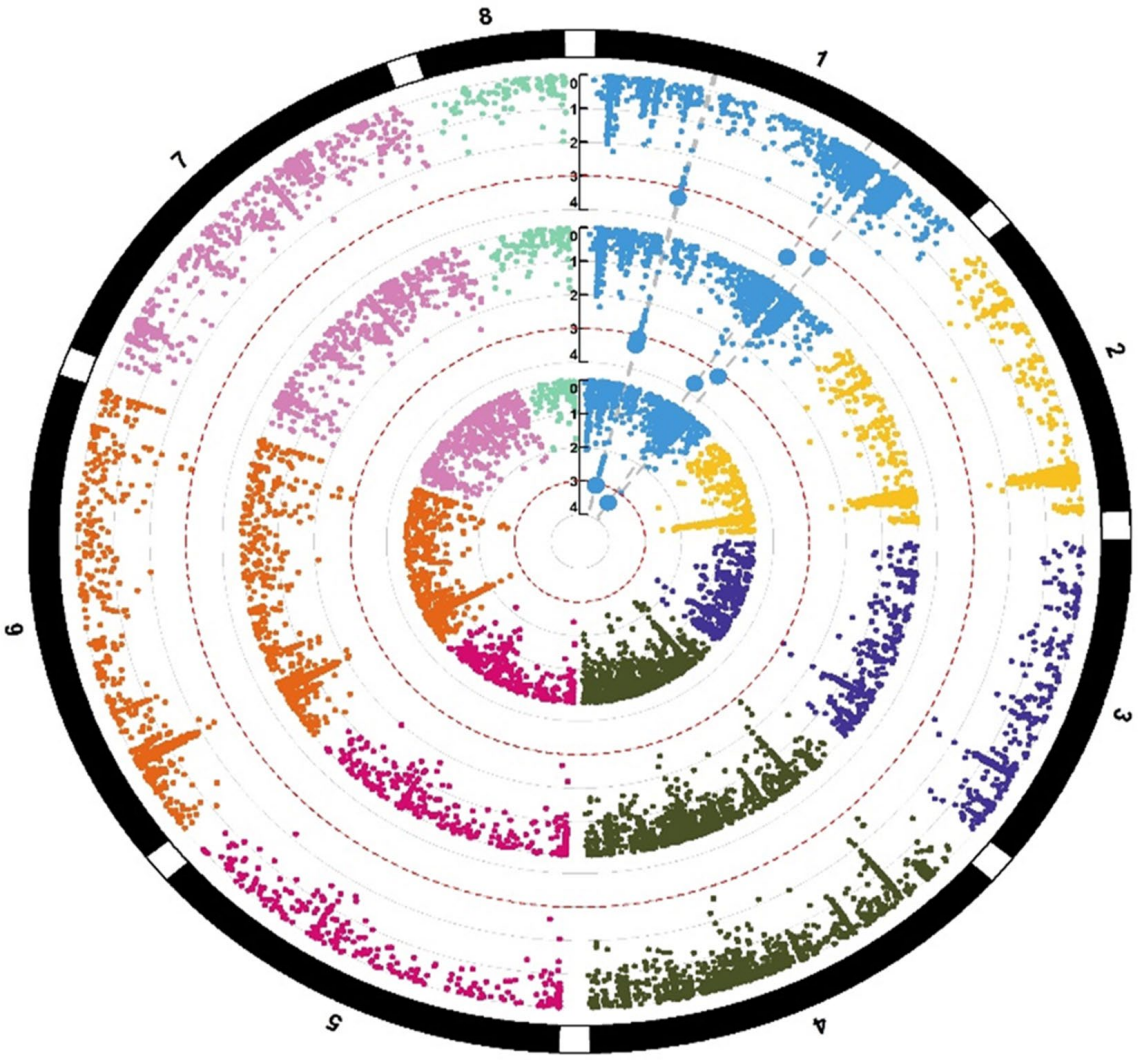
Circular Manhattan plots of showing marker-trait associations (MTAs) for seed Cu concentrations. The eight different colors represent 8 different chickpea chromosomes. The three concentric circles (each having 8 colors) shows MTAs identified for seed Cu concentrations in two environments and data pooled over environments.

In total, we identified 35 significant MTAs for all four micronutrients (P ≥ 10^–3^) on all eight pseudomolecules. The highest number of significant MTAs for micronutrients was identified on Ca6, while Ca8 contained the lowest number of MTAs. We identified 4, 3, 4, 6, 3, and 6 significant MTAs on Ca1, Ca2, Ca3, Ca4, Ca5 and Ca7 pseudomolecule SNPs, respectively (Table 5). Of these 35 SNPs, 12, 9, 9, and 5 SNPs were found to be associated with Cu, Fe, Mn, and Zn, respectively (Table 5, Figs. 1, 2, 3, 4). Interestingly, all 12 SNPs associated with Cu concentration were mapped on all eight pseudomolecules, except Ca7. Similarly, 9 SNPs associated with Fe concentration were mapped on all eight pseudomolecules, except Ca2. In the case of Mn, 9 SNPs were identified on four pseudomolecules (Ca1, Ca3, Ca4 and Ca7), while in the case of Zn concentration, 5 SNPs were identified on Ca1, Ca4 and Ca7 pseudomolecules (Table 5). The phenotypic variation explained by MTAs Zn concentration varied from 9 (Affx_123241958) to 11.6% (Affx_123247267), Fe concentration from 12.1 (Affx_123255008) to 19.9% (Affx_123275255) Cu concentration from 12.8 (Affx_123250833) to 19.6% (Affx_123255840) and Mn concentration from 4.5 (Affx_123292401) to 12.7% (Affx_123252620) (Table 5).

**Table 5:** Details of SNPs showing significant associations with seed Zn, Cu, Fe and Mn concentrations. The table shows the linkage group, position, environment of detection, and phenotypic variation explained (R2) by the marker-trait associations/SNPs identified during the present study. The table also highlighted the correspondence (validation) of some of the identified SNPs with some earlier identified genes/QTLs/SNPs for seed micronutrients.

Marker	Linkage group	Position (current study)	Environment			P value (range)	R^2^ (Range)	Trait	Comparison with earlier studies	Position mapped in earlier studies
			2017	2018	Pool					
Affx_123247267	7	29,434,198	+	−	−	2.5 × 10^–4^	11.6	Zn	–	–
Affx_123295749	7	40,625,899	+	−	−	3.3 × 10^–4^	11.3	Zn	–	–
Affx_123241958	7	42,022,431	−	+	−	7.6 × 10^–4^	9	Zn	–	–
Affx_123261732	4	34,010,123	+	+	−	4.5 × 10^–4^–8.0 × 10^–4^	9.4–10.8	Zn	–	–
Affx_123243695	1	2,896,714	−	+	−	8.3 × 10^–4^	9.4	Zn	–	–
Affx_123258734	3	27,840,598	+	−	−	2.4 × 10^–5^	14.2	Fe	–	–
Affx_123282040	4	41,579,601	+	−	−	5.8 × 10^–5^	12.9	Fe	Also reported associated with seed Fe^7^	41,702,329 and 41,702,981
Affx_123293935	6	46,127,728	+	−	−	5.8 × 10^–4^	12.8	Fe	–	–
Affx_123243960	6	58,400,703	−	+	−	1.7 × 10^–4^	14.4	Fe	–	–
Affx_123240923	6	2,398,909	+	−	−	5.9 × 10^–4^	12.8	Fe	–	–
Affx_123275255	5	12,583,357	−	+	−	3.7 × 10^–6^	19.9	Fe	–	–
Affx_123266414	7	26,236,362	−	+	−	1.2 × 10^–4^	14.8	Fe	–	–
Affx_123272321	1	2,899,986	−	+	−	2.4 × 10^–4^	13.9	Fe	Also reported associated with seed Fe^7^	3,212,701
Affx_123255008	8	9,393,627	−	+	−	8.9 × 10^–4^	12.1	Fe	–	–
Affx_123292401	7	30,961,497	+	−	+	3.8 × 10^–4^–4.0 × 10^–2^	4.5–11.3	Mn	–	–
Affx_123261947	7	3,431,242	−	+	+	5.4 × 10^–4^–2.7 × 10^–3^	8.1–9.9	Mn	–	–
Affx_123296790	3	37,944,759	+	−	+	4.4 × 10^–4^–2.5 × 10^–2^	5.2–11.1	Mn	Also reported associated with seed Mn^6^	37,831,272
Affx_123293942	3	16,499,935	−	+	+	1.7 × 10^–4^–7.9 × 10^–3^	7.9–11.5	Mn	–	–
Affx_123291734	4	44,921,315	+	−	+	4.6 × 10^–4^–5.4 × 10^–3^	7.2–11.0	Mn	–	–
Affx_123252620	4	17,033,403	−	+	+	8.0 × 10^–5^–5.5 × 10^–4^	10–12.7	Mn	–	–
Affx_123272607	4	16,986,881	−	+	+	2.5 × 10^–4^ –1.8 × 10^–3^	8.6–11.0	Mn	–	–
Affx_123296837	2	10,705,659	+	−	+	7.7 × 10^–4^–2.2 × 10^–2^	5.3–10.3	Mn	Also reported associated with seed Mn^6^	16,018,646
Affx_123282242	1	45,882,207	−	+	+	3.2 × 10^–4^–2.0 × 10^–3^	8.5–10.6	Mn	Also reported associated with seed Mn^6^	43,150,243
Affx_123280871	6	58,080,360	+	−	+	4.8 × 10^–5^–5.4 × 10^–5^	16.7–18	Cu	–	–
Affx_123294330	6	58,809,225	+	+	+	7.1 × 10^–4^–9.7 × 10^–4^	12.9–15.2	Cu	–	–
Affx_123280871	6	58,080,360	+	+	+	4.8 × 10^–5^–8.5 × 10^–5^	18.1–18.4	Cu	–	–
Affx_123294330	6	58,809,225	−	+	+	7.1 × 10^–4^–9.7 × 10^–4^	14.4–15.2	Cu	–	–
Affx_123255840	2	8,354,799	+	+	+	5.8 × 10^–5^–1.8 × 10^–4^	15.0–19.6	Cu	–	–
Affx_123299489	2	31,215,660	−	+	+	6.3 × 10^–4^–7.0 × 10^–4^	14.5–15.7	Cu	–	–
Affx_123261919	1	28,692,545	+	+	+	2.5 × 10^–4^–3.0 × 10^–4^	14.3–16.9	Cu	–	–
Affx_123252076	4	7,906,273	+	−	+	6.4 × 10^–4^–1.7 × 10^–3^	13.2–13.3	Cu	–	–
Affx_123268983	3	15,048,315	+	−	+	7.6 × 10^–4^–1.7 × 10^–3^	13.0–13.3	Cu	–	–
Affx_123250833	5	28,265,735	+	−	+	9.1 × 10^–4^–1.0 × 10^–3^	12.8–13.1	Cu	Also reported associated with seed Cu^5^	28,456,006
Affx_123251463	5	1,981,497	−	+	+	7.7 × 10^–4^–11 × 10^–3^	13.9–15.5	Cu	–	–
Affx_123259344	8	1,362,575	−	+	+	5.0 × 10^–4^–6.4 × 10^–4^	14.6–16.0	Cu	–	–

### Stable/promising and major MTAs

The MTAs identified in more than one environment were grouped as stable/promising MTAs, while MTAs with greater than 15% phenotypic variation were grouped as major MTAs. Out of the 35 identified associated SNPs, 5 SNPs were stable/promising, 6 MTAs were found to be major MTAs, and 3 MTAs were both stable and major. Among the stable/promising MTAs, 4 MTAs were for Cu (Affx_123261919, Affx_123255840, Affx_123294330 and Affx_123280871), explaining 15.6%, 17.3%, 14.05% and 18.25% of the average phenotypic variation with a P value range of 4.8 × 10^–5^ to 9.7 × 10^–4^, respectively, and one MTA (located on LG 4) for Zn (Affx_123261732, P-value = 4.5 × 10^–4^–8.0 × 10^–4^) was identified with 10.1% of the average phenotypic variation, while no MTAs were found for Fe and Mn. A set of 6 major MTAs (explaining more than 15% of phenotypic variation) were identified, of which 5 MTAs were for Cu (Affx_123259344, Affx_123261919, Affx_123255840, Affx_123280871 and Affx_123280871). The remaining major MTA (Affx_123275255) was identified for Fe, with an average phenotypic variation of 19.9%. Out of 5 major MTAs identified for seed Cu concentration, 3 MTAs (Affx_123261919, Affx_123255840 and Affx_123280871) were both stable and major. Affx_123280871 explained the maximum phenotypic variation (18.25%). The list of these markers and their chromosomal location and association with the trait of interest is presented in Table 6.

**Table 6:** Details of the stable, major, and both major & stable marker-trait associations (MTAs) identified during the present study for seed Cu, Zn,  and Fe concentration.

S. No.	Stable MTAs	Linkage group/Chromosome	Trait
1.	Affx_123261919	1	Cu
2.	Affx_123255840	2	Cu
3.	Affx_123294330	6	Cu
4.	Affx_123280871	6	Cu
5.	Affx_123261732	4	Zn
	**Major MTAs**		
1.	Affx_123259344	8	Cu
2.	Affx_123261919	1	Cu
3.	Affx_123255840	2	Cu
4.	Affx_123280871	6	Cu
5.	Affx_123280871	6	Cu
6.	Affx_123275255	5	Fe
	**Major and stable MTAs**		
1.	Affx_123261919	1	Cu
2.	Affx_123255840	2	Cu
3.	Affx_123280871	6	Cu

## Discussion

One of the major global health issues that need immediate attention is micronutrient malnutrition^38^. Iron and zinc are indispensable micronutrients in the human diet. By acting as cofactors for proteins, including hemoglobin, transcription factors and cytochrome, they play pivotal roles in growth and development. Their deficiency leads to physiological disorders such as tissue hypoxia, anemia, acrimal dermatitis, and hypogonadism. Copper plays an important role in metabolism in both plants and animals^39^. Its deficiency in the human diet causes several abnormalities in important life processes, such as collagen and elastin biosynthesis, iron mobilization, and wound healing^40^. To overcome or mitigate this problem of micronutrient malnutrition, strategies such as mineral supplementation, dietary diversification and biofortification need to be implemented^41^. Chickpea is the most important nutrient-rich grain legume crop, and incorporation of this crop in human diets is considered a very good strategy for diet diversification and alleviating micronutrient malnutrition^10–13^.

Recent advances in genomics and next-generation sequencing have enabled the availability of ample genomics resources, making crop genomics resources rich. Conventional QTL mapping approaches to identify genomic regions have been restricted to biparental mapping populations at less resolution^42^. The availability of reliable markers will help in tailoring climate-smart and nutrient-rich varieties that cater to both the demand and health aspects of humans through genomics-assisted breeding programs. The availability of genetic and genomic resources will pave the way for the discovery of useful/stable/major genes/QTLs/markers for seed micronutrients in chickpea through a variety of approaches. A variety of molecular markers have been developed in almost all crop plants and these markers have been used for variety of purposes including the study of genetic diversity, study of population structure and also in gene discovery programs^43–51^). However, despite the abundance of different types of markers available, SNPs have witnessed exponential utilization for a variety of diverse applications since their discovery^52^.

Our trait variability results indicated that the genotype × environment interactions were significant, which indicates that the significant proportion of seed micronutrient concentrations in chickpea is dependent on the soil conditions, temperature, precipitation, and various crop management practices and that significant variation also exists between the genotypes. A similar effect of location and year on seed micronutrient concentrations has been reported in rice^53^, field pea, chickpea, common bean and lentil^12^. In the present study, the concentrations of different micronutrients obtained were similar to the results observed in earlier studies^5,9–11,30,54,55^. The candidate lines identified for different micronutrients should be used in chickpea breeding programs and would also serve as useful genetic resources for gene discovery and transcriptomic studies. Diversity analysis was conducted to identify molecular genetic variations present in chickpea germplasm, revealing that indigenous chickpea genotypes are more diverse in comparison to exotic ones, which may be due to the genetic bottleneck in exotic lines or a lower number of lines included in the present study. The availability of lines carrying contrasting genetic variations with different genetic parameters will benefit breeders in chickpea breeding programs. The PCA clustered the germplasm set into two groups, indicating the existence of two subpopulations for the set of genotypes used. Similar results have been reported in some other earlier studies in chickpea making use of SSR markers^56,57^.

We report significant MTAs associated with key micronutrients through SNP data based on Axiom^®^CicerSNP Array and 2 years of phenotyping of core collections of chickpea. To optimize the results, we utilized 7% high-quality SNPs (PIC > 0.349%) after deep filtration to precisely map and identified 35 highly associated SNPs for four seed micronutrients (9 each with Fe and Mn, 5 for Zn, and 12 for Cu) in chickpea with an average of ~ 9 SNPs/trait. Quantitative trait loci for seed micronutrients have also been identified in different earlier studies in chickpea (2 QTLs for Cu^54^ and 24 QTLs for Zn and Fe^30,55^). In addition, several important genes/QTLs for different micronutrients (Zn, Fe, Cu, Mg, P, K and Mn) have also been reported in some other earlier studies in chickpea^5–7^. For seed Cu concentration, we identified significant MTAs on LG1, 2, 3, 4, 5, 6 and 8, while as MTAs were identified on LG5 and LG7 only in an earlier study^5^. For seed Zn concentrations, we identified significant MTAs on LG1, 4 and 7, while as in an earlier study, MTAs have been reported on LG4 and LG5^7^. For seed Fe concentration, we identified MTAs on LG1, 3, 4, 5, 6, 7 and 8, while as MTAs have also been reported for seed Fe concentrations on LG1,4 and LG5 in an earlier study^7^. For Mn concentration, we identified significant MTAs on LG1, 2, 3, 4 and 7, while MTAs have been reported on LG1, 2, 3, 4, 6 and 7 in an earlier study^6^. The comparison of the results revealed that several unique and new MTAs on different chromosomes not reported on these chromosomes in earlier studies have been reported during the present study (Table 5). On the other hand, a set of six MTAs (2 for Fe, 3 for Mn and 1 for Cu) reported by us during the present study have been also reported in the same/almost same genomic regions in earlier studies^5–7^ (Table 5). Therefore, these MTAs have been declared as validated MTAs. All 35 SNPs identified during the present study were detected on all eight chickpea linkage groups/chromosomes (LG1–LG8). Genomic regions identified in the present study have also been flanked by QTLs responsible for seed micronutrients in chickpea; for example, QTLs for seed Fe were present on Ca1, Ca3, Ca4, Ca5 and Ca7^10,30,54^. This could be due to the different genetic backgrounds of the germplasm used in our study. The present study also identified QTLs at different chromosomal positions not reported earlier, suggesting that we have identified novel QTLs linked to seed micronutrients in chickpea. QTLs for all four micronutrients have also been mapped in different crops, including lentil for Zn (on chromosome 1, 2, 3, and 4), and Fe (on chromosome 3, 5 and 6)^58^; pea for Zn (on chromosome 2, 3, 5 and 7), Fe (on chromosome 2, 3, 5, 6 and 7), and Mn (on chromosome 1, 2, 4, 5, 6 and 7)^59^; and *Medicago truncatula* for Zn (on chromosomes 1, 2, 3, 4, 5, and 7), Fe (on chromosomes 7 and 8), Cu (on chromosome 1, 2, 4 and 7), and Mn (on chromosomes 1, 2, 3, 5, 6 and 7)^60^. The markers found to have a significant association with the trait should be used in marker-assisted selection for biofortified chickpea to mitigate the problem of micronutrient malnutrition or hidden hunger.

## Conclusions

Our study is the first of its kind that presents the features of a large population of chickpea genotypes in different environments grown in the region of Kashmir, providing an understanding of the influence of environmental and growing conditions on the seed micronutrient concentrations of a crop. This study provides insight into the genetic basis of variability for seed micronutrient concentrations in chickpea and the potential of GWAS in unravelling marker trait associations in economically important crops. Because of their importance, stable MTAs should be recommended for chickpea molecular breeding programs aimed at enhancing the seed micronutrient concentrations of local chickpea landraces preferred by consumers. The stable and major MTAs identified during the present study will prove useful in breeding programs aimed at enhancing seed micronutrient concentrations in chickpea.

## Methods

The plant material used, trait data recorded, genotyping done and data analysis conducted in this study has been presented under separate headings below. All the methods in this manuscript were carried out in accordance with relevant guidelines and regulations.

### Plant material

We prepared a chickpea core set comprising 147 genotypes (128 Desi, 12 Kabuli and 7 intermediate pea shaped) based on SSR marker data generated on 384 composite sets^56^ (Supplementary Table S3). The germplasm lines were obtained from the International Crops Research Institute for Semi-arid Tropics (ICRISAT) Hyderabad, Indian Council of Agricultural Research-Indian Institute of Pulse Research (ICAR-IIPR), Kanpur, and National Bureau of Plant Genetic Resources (NBPGR), New Delhi and Rafi Ahmad Kidwai, RAK College of Agriculture, Rajmata Vijayaraje Scindia Krishi Vishwa Vidyalaya, Sehore, Madhya Pradesh. However, it is important to mention that “The material was not collected by us but was procured from the national/international institutions after signing a proper Material Transfer Agreement (MTA) with these institutions”.

### Trait phenotyping

Trait phenotyping to quantify the seed micronutrient concentration for four micronutrients Zn, Fe, Cu, and Mn was performed using the core set, grown in two replicated field trials arranged in an augmented block design with a planting density of 10 seeds per row per genotype in rows of 4 m long with a row-to-row spacing of 30 cm. The trial was conducted for two consecutive years, 2017 and 2018, in the research fields of the Faculty of Agriculture (FoA), SKUAST-K, Wadura Campus, Sopore, Kashmir, India. The concentrations of four micronutrients, Zn, Fe, Cu, and Mn, were determined following the procedure described by Ref.^61^. Healthy mature harvested seeds from each genotype, free from any impurity, were surface cleaned with double-distilled water and dried in an oven at 55–60 °C for 48 h. The dry seeds were ground into powder using a ball mill (MM400). For each genotype, one gram of powder was weighed in an Erlenmeyer flask, and acid digestion was conducted on a hot plate (250 °C) by adding 10 ml of HNO_3_-HClO_4_ (9:4) solution to each flask^61^. The digested mixture was filtered through Whatman No. 40 filter paper, and using double distilled water, the total volume was made up to 50 ml. The chemicals used in the digestion of the samples were of analytical grade. The samples were analyzed against standards of known concentration. The concentrations of nutrients were determined using an atomic absorption spectrophotometer (AAS) (Agilent Technologies, 200 series). The concentrations of micronutrients present were estimated and expressed as mg/kg (ppm) of seed. Three independent replications were noted for each accession, and their mean was used for the statistical analysis.

### Genome-wide marker genotyping/whole-genome scanning

#### DNA extraction and purification

The genomic DNA of 147 chickpea genotypes was isolated and purified using a high-throughput DNeasy Plant Mini Kit by Qiagen (www.qiagen.com/handbooks) following the manufacturer’s instructions. For DNA isolation, leaf samples from three random healthy plants for each genotype were taken. After purification, the DNA was dissolved in 100 μl Tris–EDTA (TE) buffer. A NanoDrop (ND 1000) was used for the quantification of the isolated DNA samples, and the purity of the samples was analyzed by agarose gel electrophoresis using 0.8% agarose gel in the presence of Tris–acetate-EDTA (1× TAE) buffer.

#### SNP genotyping using 50K SNP Axiom Cicer SNP Array

The purified high-quality DNA (100 ng µl^−1^) from a set of 147 chickpea genotypes was further used for genotyping using 50K SNP array^37^ at the Centre of Excellence in Genomics and Systems Biology (CEGSB) Lab Facilities, ICRISAT, Patancheru, Hyderabad. The total genomic DNA before hybridization was fragmented (25–125 bp) followed by purification and hybridized with the 50K SNP Axiom CicerSNP Array to generate the genotyping data. The nonspecific random ligations bound to the targets were washed off under stringent conditions. The Gene Titan-Multi-Channel instrument was used to stain, image and process the array to track to the multicolor ligation events at the array surface, which points toward the polymorphic nucleotides and ultimately generate the data.

### Data analysis

#### Analysis of variance

Based on the mixed-effect model for three different replications and 2 years using the statistical functions package of R software according to 5% significance levels, analysis of variance (ANOVA) was conducted to analyze differences in the seed nutrient concentrations. The diverse statistical measures, including minimum, maximum, average value, standard deviation and coefficient of variation (CV), among the diverse accessions were measured. The effect of genotype, environment (year) and their combined effect (G × E) was also analysed. Levene’s test was also conducted to test the homogeneity of variance of genotypes in two different environments/experiments using an established procedure in Microsoft Excel (https://www.youtube.com/watch?v=HoYos9IwZNY). The test is used to test whether the trait data collected in two different experiments/environments are significantly different. A P value < 0.05 indicates significant differences between the variances (heterogeneous variances), while P > 0.05 indicates nonsignificant differences between the trait values of two experiments (homogenous variances).

#### Diversity analysis and population differentiation

Diversity analysis was performed using genotypic data generated by a set of 7277 SNPs on 147 chickpea genotypes. The software program GenAlEx version 6.5^62^ was utilized to statistically analyze the genotypic data, and parameters such as the total number of alleles (Na), the number of effective alleles (Ne) and Shannon’s information index were calculated. To conduct principal coordinate analysis (PCA), the genotypic data (multiple loci and multiple genotypes) were also analyzed by GenAlEx version 6.5 software. For the construction of the PCA plots, a pairwise genetic distance matrix was calculated. The same software program GenAlEx version 6.5 was used to compute the other genetic diversity parameters, such as genetic identity, gene flow (Nm), and pairwise Nei’s unbiased genetic distance. Using the software package Power-Marker version 3.25^63^, we calculated the polymorphic information content (PIC) for each SNP marker locus.

#### Analysis of molecular variance (AMOVA) and genetic diversity indices

To calculate the level of genetic variation not only among the genotypes but also within the populations, analysis of molecular variance (AMOVA) was performed. The genetic distance matrix (same as used for PCA) was utilized for conducting AMOVA. The software program GenAlEx6.5 was utilized to perform the AMOVA.

#### Genome-wide association analyses

**Figure 5:**
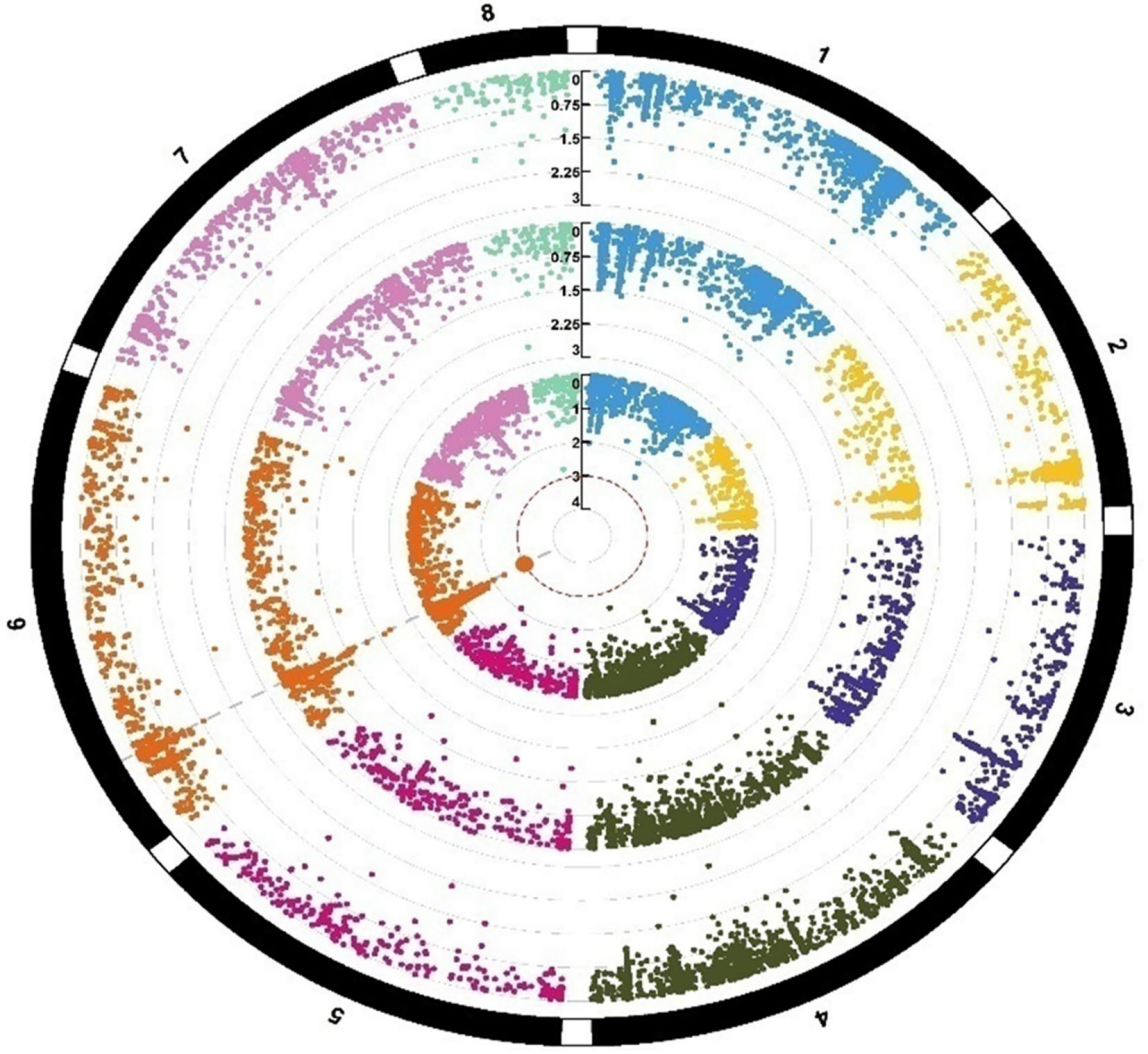
Circular Manhattan plots of showing marker-trait associations (MTAs) for seed Fe concentrations. The eight different colors represent 8 different chickpea chromosomes. The three concentric circles (each having 8 colors) shows MTAs identified for seed Fe concentrations in two environments and data pooled over environments.

**Figure 6:**
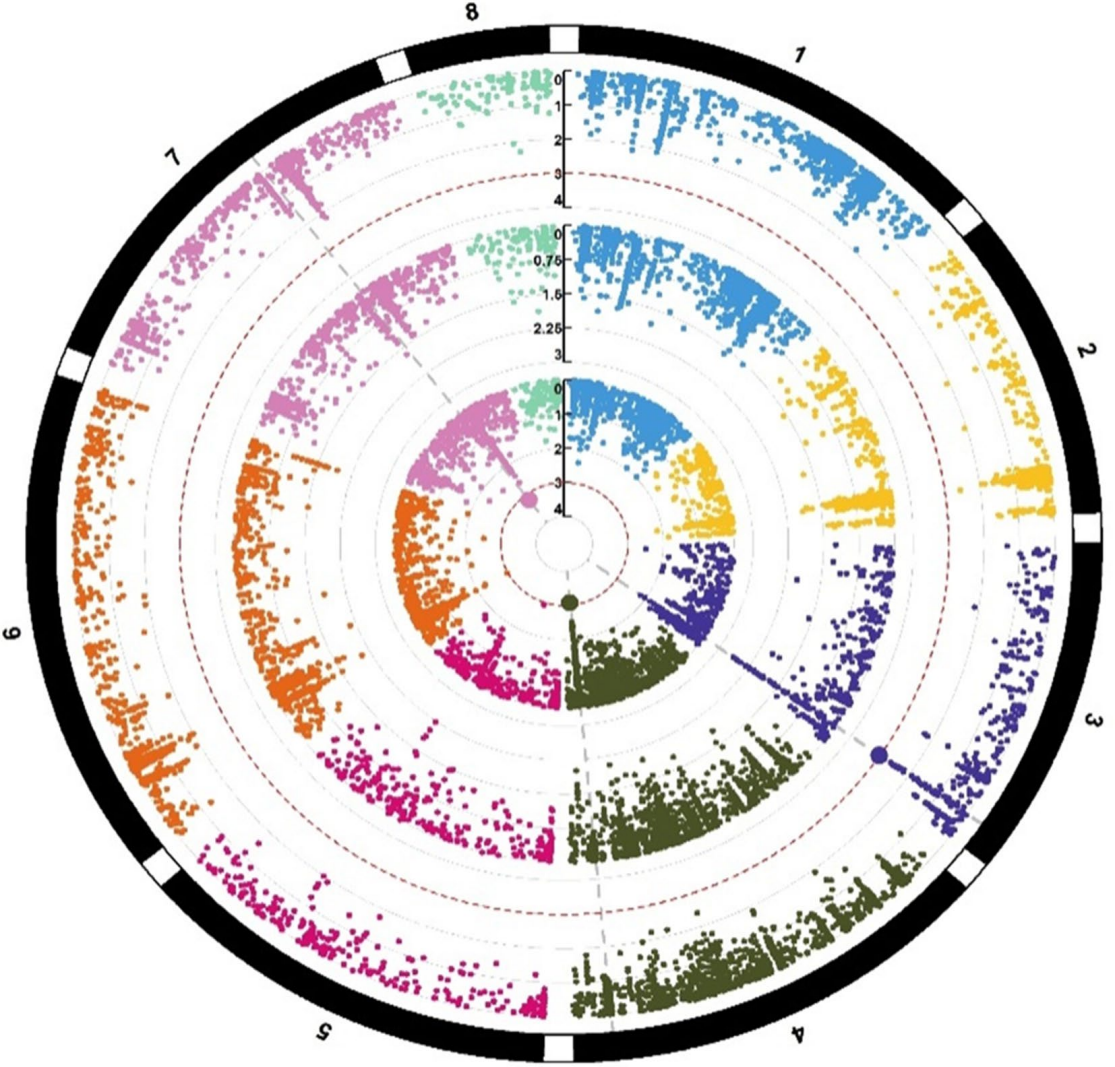
Circular Manhattan plots of showing marker-trait associations (MTAs) for seed Mn concentrations. The eight different colors represent 8 different chickpea chromosomes. The three concentric circles (each having 8 colors) shows MTAs identified for seed Mn concentrations in two environments and data pooled over environments.

**Figure 7:**
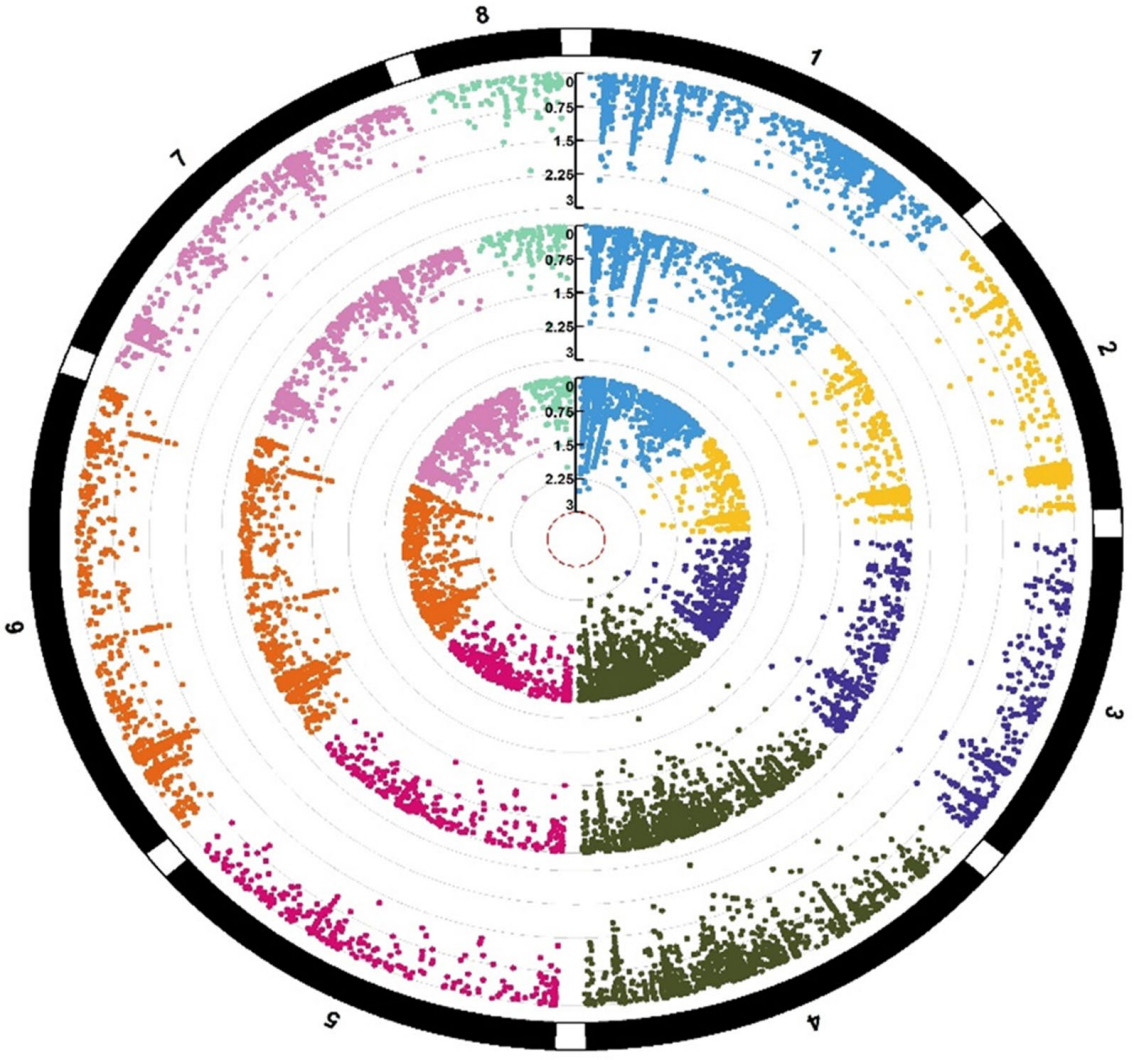
Circular Manhattan plots of showing marker-trait associations (MTAs) for seed Zn concentrations. The eight different colors represent 8 different chickpea chromosomes. The three concentric circles (each having 8 colors) shows MTAs identified for seed Zn concentrations in two environments and data pooled over environments.

To identify markers associated with seed Zn, Fe, Cu, and Mn concentrations, agenome-wide association study (GWAS) was conducted. GAPIT (The Genome Association and Prediction Integrated Tool) version 3.0, R package (http://zzlab.net/GAPIT) was used for analyzing the trait phenotyping data (concentration of micronutrients analyzed through AAS) and genotyping data (obtained through the use of Axiom Cicer SNP Array). The model used in GWAS was MLM (mixed linear model), as it has proven quite useful for controlling the relatedness and population structure within GWAS. The population structure in MLM is considered a fixed effect, whereas kinship among individuals is incorporated as the variance–covariance structure of the random effect for the individuals^64^. SNPs showing minor allele frequencies below 0.05 were eliminated from the dataset. In total, 41,489 polymorphic markers were retained for the 147 genotyped accessions. To show the distribution of the P-values for the SNP markers, Manhattan plots were generated (Figs. 4, 5, 6, 7). The significant association between SNP and trait of interest was declared at a threshold value of P (≥ 10^–3^).

### Declaration

All the methods in this manuscript were carried out in accordance with relevant guidelines and regulations.

## Supplementary Information

Supplementary Figures.
