# Supplementary material for: Genome-wide association analysis to delineate high-quality SNPs for seed micronutrient density in chickpea (Cicer arietinum L.)
Source: Sci Rep. 2022 Sep 5;12:11357. doi: 10.1038/s41598-022-14487-1 (PMC9445022; doi:10.1038/s41598-022-14487-1)
Supplement: Supplementary file 1 — Supplementary Figures. [file 41598_2022_14487_MOESM1_ESM.docx]

**Genome-wide association analysis to delineate high-quality SNPs for seed micronutrient density in chickpea (*Cicer arietinum* L.)**

**Humara Fayaz^1,2#^, Sandhya Tyagi^3#^, Aijaz A. Wani^2^, Renu Pandey^3^, Sabina Akhtar^4^, Mohd Ashraf Bhat^1^, Annapurna Chitikineni^5^, Rajeev Kumar Varshney^5,6^, Mahendar Thudi^5,7,8*^, Upendra Kumar^9^, Reyazul Rouf Mir^1*^**

**Supplementary Table S1:** Summary of AMOVA

| **Source** | **df** | **SS** | **MS** | **Est. Var.** | **% Variation** |
| --- | --- | --- | --- | --- | --- |
| **Among Pops** | 1 | 16548.168 | 16548.168 | 517.472 | 7% |
| **Within Pops** | 145 | 1000903.043 | 6902.780 | 6902.780 | 93% |
| **Total** | 146 | 1017451.211 |  | 7420.251 | 100% |

**Supplementary Table S2:** Number of identified loci with private alleles in different samples.

| **Sample** | **Population** | **No. Loci with Private Alleles** | **Sample** | **Population** | **No. Loci with Private Alleles** |
| --- | --- | --- | --- | --- | --- |
| **CC1** | **2** | **363** | **CC152** | **1** | **348** |
| **CC102** | **1** | **377** | **CC154** | **1** | **351** |
| **CC107** | **1** | **358** | **CC155** | **1** | **360** |
| **CC11** | **2** | **348** | **CC16** | **2** | **390** |
| **CC111** | **1** | **361** | **CC160** | **1** | **398** |
| **CC113** | **1** | **395** | **CC162** | **1** | **342** |
| **CC114** | **1** | **350** | **CC168** | **1** | **340** |
| **CC117** | **1** | **388** | **CC171** | **1** | **360** |
| **CC12** | **2** | **376** | **CC175** | **1** | **361** |
| **CC120** | **1** | **377** | **CC179** | **1** | **368** |
| **CC121** | **1** | **380** | **CC180** | **1** | **354** |
| **CC122** | **1** | **367** | **CC184** | **1** | **379** |
| **CC126** | **1** | **398** | **CC187** | **1** | **389** |
| **CC130** | **1** | **337** | **CC188** | **1** | **384** |
| **CC131** | **1** | **338** | **CC189** | **1** | **370** |
| **CC133** | **1** | **368** | **CC191** | **1** | **389** |
| **CC14** | **2** | **359** | **CC192** | **1** | **372** |
| **CC140** | **1** | **362** | **CC193** | **1** | **367** |
| **CC144** | **1** | **378** | **CC196** | **1** | **378** |
| **CC145** | **1** | **361** | **CC198** | **1** | **365** |
| **CC146** | **1** | **382** | **CC199** | **1** | **373** |
| **CC147** | **1** | **359** | **CC27** | **1** | **362** |
| **CC149** | **1** | **379** | **CC29** | **1** | **379** |
| **CC150** | **1** | **366** | **CC31** | **1** | **380** |
| **CC32** | **1** | **355** | **CP16** | **1** | **44** |
| **CC34** | **1** | **356** | **CP19** | **1** | **61** |
| **CC37** | **1** | **373** | **CP2** | **1** | **74** |
| **CC38** | **1** | **362** | **CP23** | **2** | **74** |
| **CC39** | **1** | **364** | **CP24** | **2** | **59** |
| **CC43** | **1** | **363** | **CP25** | **1** | **59** |
| **CC48** | **1** | **379** | **CP27** | **1** | **54** |
| **CC49** | **1** | **346** | **CP28** | **1** | **70** |
| **CC5** | **2** | **357** | **CP4** | **1** | **51** |
| **CC50** | **1** | **370** | **CP5** | **1** | **72** |
| **CC52** | **1** | **346** | **CP6** | **1** | **66** |
| **CC57** | **1** | **349** | **CP9** | **1** | **67** |
| **CC59** | **1** | **354** | **H1** | **1** | **34** |
| **CC61** | **1** | **354** | **H10** | **1** | **48** |
| **CC63** | **1** | **362** | **H12** | **1** | **60** |
| **CC65** | **1** | **369** | **H13** | **1** | **69** |
| **CC66** | **1** | **381** | **H14** | **1** | **39** |
| **CC74** | **1** | **363** | **H16** | **1** | **37** |
| **CC76** | **1** | **352** | **H3** | **1** | **38** |
| **CC77** | **1** | **355** | **H4** | **1** | **47** |
| **CC83** | **1** | **366** | **H5** | **1** | **28** |
| **CC87** | **1** | **343** | **H8** | **1** | **58** |
| **CC88** | **1** | **337** | **H9** | **1** | **28** |
| **CC9** | **2** | **352** | **K21** | **1** | **58** |
| **CC92** | **1** | **363** | **K30** | **1** | **49** |
| **CC96** | **1** | **377** | **K32** | **1** | **60** |
| **CC98** | **1** | **360** | **K35** | **1** | **53** |
| **K40** | **1** | **66** | **NK59** | **1** | **67** |
| **K42** | **1** | **59** | **NK63** | **1** | **83** |
| **NK1** | **1** | **51** | **NK65** | **1** | **71** |
| **NK10** | **1** | **49** | **NK7** | **1** | **74** |
| **NK11** | **1** | **75** | **NK70** | **1** | **361** |
| **NK112** | **1** | **51** | **NK75** | **1** | **71** |
| **NK116** | **1** | **70** | **NK88** | **1** | **70** |
| **NK124** | **1** | **352** | **NK9** | **1** | **44** |
| **NK2** | **1** | **70** | **S21** | **2** | **46** |
| **NK21** | **1** | **66** | **Y1** | **1** | **30** |
| **NK22** | **1** | **46** | **Y10** | **1** | **34** |
| **NK23** | **1** | **58** | **Y11** | **1** | **42** |
| **NK31** | **1** | **59** | **Y12** | **1** | **30** |
| **NK35** | **1** | **56** | **Y14** | **1** | **46** |
| **NK41** | **1** | **76** | **Y15** | **1** | **37** |
| **NK42** | **1** | **60** | **Y16** | **1** | **81** |
| **NK48** | **1** | **58** | **Y17** | **1** | **50** |
| **NK55** | **1** | **386** |  |  |  |

**Supplementary Table S3:** List of germplasm used, source provided the material and type/color of seed of particular genotype.

| **S.No** | **Assigned number** | **Accession number** | **Source** | **Seed type/Color** |
| --- | --- | --- | --- | --- |
| 1 | CC1 | EC 3507 | NBPGR-NEW DELHI | Desi/Brown |
| 2 | CC102 | IC 327868 | NBPGR-NEW DELHI | Desi/Green |
| 3 | CC107 | IC 328046 | NBPGR-NEW DELHI | Pea/Beige |
| 4 | CC11 | EC 538493 | NBPGR-NEW DELHI | Desi/Brown |
| 5 | CC111 | IC 348453 | NBPGR-NEW DELHI | Pea/Beige |
| 6 | CC113 | IC 348481 | NBPGR-NEW DELHI | Desi/Black |
| 7 | CC114 | IC 348499 | NBPGR-NEW DELHI | Desi/ Blakish brown |
| 8 | CC117 | IC 376248 | NBPGR-NEW DELHI | Desi/Brown |
| 9 | CC12 | EC 548032 | NBPGR-NEW DELHI | Desi/Brown |
| 10 | CC120 | IC 396762 | NBPGR-NEW DELHI | Desi/Brown |
| 11 | CC121 | IC 408004 | NBPGR-NEW DELHI | Desi/Brown |
| 12 | CC122 | IC 408075 | NBPGR-NEW DELHI | Desi/Brown |
| 13 | CC126 | IC 408261 | NBPGR-NEW DELHI | Desi/Brown |
| 14 | CC130 | IC 415651 | NBPGR-NEW DELHI | Desi/Black |
| 15 | CC131 | IC 424251 | NBPGR-NEW DELHI | Desi/Brown |
| 16 | CC133 | IC 446511 | NBPGR-NEW DELHI | Desi/Black |
| 17 | CC14 | EC 555205 | NBPGR-NEW DELHI | Desi/Brown |
| 18 | CC140 | IC 485688 | NBPGR-NEW DELHI | Desi/Brown |
| 19 | CC144 | IC 486365 | NBPGR-NEW DELHI | Desi/Brown |
| 20 | CC145 | IC 486759 | NBPGR-NEW DELHI | Desi/Brown |
| 21 | CC146 | IC 486809 | NBPGR-NEW DELHI | Desi/Brown |
| 22 | CC147 | IC 486818 | NBPGR-NEW DELHI | Desi/Brown |
| 23 | CC149 | IC 486922 | NBPGR-NEW DELHI | Desi/Brown |
| 24 | CC150 | IC 486952 | NBPGR-NEW DELHI | Desi/Brown |
| 25 | CC152 | IC 486965 | NBPGR-NEW DELHI | Desi/Brown |
| 26 | CC154 | IC 486996 | NBPGR-NEW DELHI | Desi/Brown |
| 27 | CC155 | IC 486997 | NBPGR-NEW DELHI | Desi/Beige |
| 28 | CC16 | EC 555299 | NBPGR-NEW DELHI | Desi/Black |
| 29 | CC160 | IC 487126 | NBPGR-NEW DELHI | Desi/Brown |
| 30 | CC162 | IC 487193 | NBPGR-NEW DELHI | Desi/Brown |
| 31 | CC168 | IC 489883 | NBPGR-NEW DELHI | Desi/Brown |
| 32 | CC171 | IC 522130 | NBPGR-NEW DELHI | Desi/Black |
| 33 | CC175 | IC 552192 | NBPGR-NEW DELHI | Desi/Brown |
| 34 | CC179 | IC 567580 | NBPGR-NEW DELHI | Desi/Brown |
| 35 | CC180 | IC 587382 | NBPGR-NEW DELHI | Desi/Brown |
| 36 | CC184 | IC 0486625 | NBPGR-NEW DELHI | Desi/Brown |
| 37 | CC187 | EC 532435 | NBPGR-NEW DELHI | Desi/Black |
| 38 | CC188 | EC 532477 | NBPGR-NEW DELHI | Desi/Blakish brown |
| 39 | CC189 | IC 118913 | NBPGR-NEW DELHI | Desi/Beige |
| 40 | CC191 | IC 269630 | NBPGR-NEW DELHI | Desi/Brown |
| 41 | CC192 | IC 270707 | NBPGR-NEW DELHI | Desi/Brown |
| 42 | CC193 | IC 272089 | NBPGR-NEW DELHI | Desi/Brown |
| 43 | CC196 | IC 272459 | NBPGR-NEW DELHI | Desi/Brown |
| 44 | CC198 | IC 272672 | NBPGR-NEW DELHI | Desi/Brown |
| 45 | CC199 | IC 275637 | NBPGR-NEW DELHI | Desi/Brown |
| 46 | CC27 | IC 83411 | NBPGR-NEW DELHI | Desi/Brown |
| 47 | CC29 | IC 83660 | NBPGR-NEW DELHI | Desi/Brown |
| 48 | CC31 | IC 83729 | NBPGR-NEW DELHI | Desi/Brown |
| 49 | CC32 | IC 83959 | NBPGR-NEW DELHI | Desi/Green |
| 50 | CC34 | IC 95076 | NBPGR-NEW DELHI | Desi/Brown |
| 51 | CC37 | IC 116384 | NBPGR-NEW DELHI | Desi/Brown |
| 52 | CC38 | IC 116783 | NBPGR-NEW DELHI | Desi/Beige |
| 53 | CC39 | IC 117673 | NBPGR-NEW DELHI | Desi/Brown |
| 54 | CC43 | IC 172324 | NBPGR-NEW DELHI | Desi/Brown |
| 55 | CC48 | IC 209240 | NBPGR-NEW DELHI | Desi/Blakish brown |
| 56 | CC49 | IC 209243 | NBPGR-NEW DELHI | Desi/Brown |
| 57 | CC5 | EC 442024 | NBPGR-NEW DELHI | Desi/Brown |
| 58 | CC50 | IC 209285 | NBPGR-NEW DELHI | Desi/Brown |
| 59 | CC52 | IC 209463 | NBPGR-NEW DELHI | Desi/Cream |
| 60 | CC57 | IC 244433 | NBPGR-NEW DELHI | Desi/Brown |
| 61 | CC59 | IC 265298 | NBPGR-NEW DELHI | Desi/Brown |
| 62 | CC61 | IC 269123 | NBPGR-NEW DELHI | Desi/Brown |
| 63 | CC63 | IC 269161 | NBPGR-NEW DELHI | Desi/Brown |
| 64 | CC65 | IC 269404 | NBPGR-NEW DELHI | Desi/Brown |
| 65 | CC66 | IC 269413 | NBPGR-NEW DELHI | Kabuli/Beige |
| 66 | CC74 | IC 271940 | NBPGR-NEW DELHI | Desi/Brown |
| 67 | CC76 | IC 272471 | NBPGR-NEW DELHI | Desi/Brown |
| 68 | CC77 | IC 272650 | NBPGR-NEW DELHI | Desi/Brown |
| 69 | CC83 | IC 275563 | NBPGR-NEW DELHI | Desi/Brown |
| 70 | CC87 | IC 296163 | NBPGR-NEW DELHI | Desi/Brown |
| 71 | CC88 | IC 297528 | NBPGR-NEW DELHI | Desi/Beige |
| 72 | CC9 | EC 498825 | NBPGR-NEW DELHI | Pea/Beige |
| 73 | CC92 | IC 305487 | NBPGR-NEW DELHI | Desi/Brown |
| 74 | CC96 | IC 308562 | NBPGR-NEW DELHI | Desi/Brown |
| 75 | CC98 | IC 327528 | NBPGR-NEW DELHI | Desi/Brown |
| 76 | CP16 | ICC 13077 | ICRISAT | Desi/Green |
| 77 | CP19 | ICC 7537 | ICRISAT | Desi/Green |
| 78 | CP2 | ICC 8245 | ICRISAT | Desi/Green |
| 79 | CP23 | GSC 7 | ICRISAT | Desi/Green |
| 80 | CP24 | GCS 5 | ICRISAT | Desi/Green |
| 81 | CP25 | JGC1 | ICRISAT | Desi/Brown |
| 82 | CP27 | ICCV 96029 | ICRISAT | Desi/Beige |
| 83 | CP28 | ICC 16207 | ICRISAT | Desi/Brown |
| 84 | CP4 | ICC 8243 | ICRISAT | Desi/Green |
| 85 | CP5 | ICC 6814 | ICRISAT | Desi/Green |
| 86 | CP6 | ICC 8504 | ICRISAT | Desi/Brown |
| 87 | CP9 | ICC 4969 | ICRISAT | Desi/Green |
| 88 | H1 | ICC 11284 | ICRISAT | Kabuli/Cream |
| 89 | H10 | ICC 96030 | ICRISAT | Desi/Brown |
| 90 | H12 | ICC 12424 | ICRISAT | Desi/Brown |
| 91 | H13 | ICC 5810 | ICRISAT | Desi/Black |
| 92 | H14 | ICC 6306 | ICRISAT | Desi/Black |
| 93 | H16 | ICC 11180 | ICRISAT | Desi/Brown |
| 94 | H3 | ICC 12968 | ICRISAT | Kabuli/Beige |
| 95 | H4 | ICC 8927 | ICRISAT | Kabuli/Beige |
| 96 | H5 | KAK 2 | ICRISAT | Kabuli/Beige |
| 97 | H8 | ICC 16349 | ICRISAT | Desi/Brown |
| 98 | H9 | JG 11 | ICRISAT | Desi/Brown |
| 99 | K21 | ICC 10956 | IIPR Kanpur | Desi/Brown |
| 100 | K30 | ICC 15019 | IIPR Kanpur | Desi/Brown |
| 101 | K32 | ICC 15089 | IIPR Kanpur | Desi/Brown |
| 102 | K35 | ICC 14245 | IIPR Kanpur | Desi/Brown |
| 103 | K40 | ICC 15850 | IIPR Kanpur | Desi/Brown |
| 104 | K42 | ICC 12909 | IIPR Kanpur | Desi/Brown |
| 105 | NK1 | ICC 10400 | IIPR Kanpur | Desi/Brown |
| 106 | NK10 | ICC 15201 | IIPR Kanpur | Desi/Brown |
| 107 | NK11 | ICC 4567 | IIPR Kanpur | Desi/Brown |
| 108 | NK112 | JG 74 | IIPR Kanpur | Desi/Brown |
| 109 | NK116 | ICC 15198 | IIPR Kanpur | Desi/Brown |
| 110 | NK124 | ICC 9239 | IIPR Kanpur | Desi/Brown |
| 111 | NK2 | ICC 12948 | IIPR Kanpur | Desi/Brown |
| 112 | NK21 | ICC 15058 | IIPR Kanpur | Desi/Brown |
| 113 | NK22 | ICC 11091 | IIPR Kanpur | Desi/Brown |
| 114 | NK23 | ICC 14729 | IIPR Kanpur | Desi/Brown |
| 115 | NK31 | ICC 12354 | IIPR Kanpur | Desi/Brown |
| 116 | NK35 | ICC 15177 | IIPR Kanpur | Desi/Brown |
| 117 | NK41 | ICC 14843 | IIPR Kanpur | Desi/Brown |
| 118 | NK42 | ICC 12299 | IIPR Kanpur | Desi/Brown |
| 119 | NK48 | C-235 | IIPR Kanpur | Desi/Brown |
| 120 | NK55 | ICC-11224 | IIPR Kanpur | Desi/Brown |
| 121 | NK59 | JG-218 | IIPR Kanpur | Desi/Brown |
| 122 | NK63 | IPC-09-58 | IIPR Kanpur | Desi/Brown |
| 123 | NK65 | ICC 16684 | IIPR Kanpur | Desi/Brown |
| 124 | NK7 | ICC 4431 | IIPR Kanpur | Desi/Brown |
| 125 | NK70 | ICC 15921 | IIPR Kanpur | Desi/Brown |
| 126 | NK75 | ICC 15033 | IIPR Kanpur | Desi/Brown |
| 127 | NK88 | PUSA 391 | IIPR Kanpur | Desi/Green |
| 128 | NK9 | ICC 10498-B | IIPR Kanpur | Desi/Brown |
| 129 | S21 | Shalimar Chickpea | SKAUST-K | Kabuli/Beige |
| 130 | Y1 | RVSSG 32 | RAK-college-M.P | Desi/Green |
| 131 | Y10 | AGBL-P-S-170008 | RAK-college-M.P | Pea/Beige |
| 132 | Y11 | AGBL-D-170009 | RAK-college-M.P | Desi/Brown |
| 133 | Y12 | AGBL-D-170010 | RAK-college-M.P | Desi/Brown |
| 134 | Y14 | AGBL-D-170012 | RAK-college-M.P | Desi/Brown |
| 135 | Y15 | AGBL-D-170013 | RAK-college-M.P | Kabuli/Cream |
| 136 | Y16 | RVSSG 30 | RAK-college-M.P | Kabuli/Cream |
| 137 | Y17 | RVSSG 37 | RAK-college-M.P | Kabuli/Cream |
| 138 | Y18 | RVSSG 47 | RAK-college-M.P | Kabuli/Beige |
| 139 | Y19 | RVSSG 46 | RAK-college-M.P | Kabuli/Cream |
| 140 | Y2 | AGBL-G-170001 | RAK-college-M.P | Desi/Green |
| 141 | Y20 | AGBL-K-170014 | RAK-college-M.P | Kabuli/Cream |
| 142 | Y3 | AGBL-G-170002 | RAK-college-M.P | Desi/Green |
| 143 | Y4 | AGBL-G-170003 | RAK-college-M.P | Desi/Green |
| 144 | Y5 | AGBL-G-170004 | RAK-college-M.P | Desi/Green |
| 145 | Y7 | AGBL-P-S-170006 | RAK-college-M.P | Pea/Beige |
| 146 | Y8 | AGBL-P-S-170007 | RAK-college-M.P | Pea/Beige |
| 147 | Y9 | RVSSG 44 | RAK-college-M.P | Pea/Beige |
